# Individual and Environmental Factors Associated with Tobacco Smoking, Alcohol Abuse and Illegal Drug Consumption in University Students: A Mediating Analysis

**DOI:** 10.3390/ijerph17093019

**Published:** 2020-04-27

**Authors:** Laura Delgado-Lobete, Rebeca Montes-Montes, Alba Vila-Paz, José-Manuel Cruz-Valiño, Berta Gándara-Gafo, Miguel-Ángel Talavera-Valverde, Sergio Santos-del-Riego

**Affiliations:** 1 Health Integration and Promotion Research Unit (INTEGRA SAÚDE), Faculty of Health Sciences, University of A Coruña, 15006 A Coruña, Spain; 2 TALIONIS Research Group, Faculty of Health Sciences, University of A Coruña, 15008 A Coruña, Spain; 3 Institute for Oral Implantology and Rehabilitation of A Coruña (Instituto Coruñés de Implantología y Rehabilitación Oral-ICIRO), 15007 A Coruña, Spain; 4 UDC Saudable, Health Promotion Department of University of A Coruña, 15011 A Coruña, Spain

**Keywords:** university students, substance abuse, alcohol abuse, smoking, tobacco, drug use, environmental factors, mediating analysis

## Abstract

Substance abuse is a major and prevalent public health concern among university students. Tobacco smoking, risky alcohol behavior, and illegal drug consumption may lead to health problems and behavioral and academic issues. Several individual and environmental factors associate with substance abuse in this population, and the mediating effect of alcohol abuse in the relationship between tobacco smoking and drug consumption is yet to be explored. The purposes of this study were to evaluate the association of individual and environmental factors and substance use, and to analyze the relationship between tobacco smoking, alcohol abuse, and drug consumption, considering alcohol abuse as a possible mediator. A total of 550 Spanish undergraduate and postgraduate students completed several questionnaires regarding their smoking status, alcohol use, and drug consumption during the last six months. Bivariate and multivariate analyses were conducted to explore associations between factors. Direct, indirect and mediating effects were tested using a partial least squares approach (PLS-SEM). The results indicated that substance abuse is associated with being male, living with other students, and combined substance consumption. PLS-SEM showed a significant effect of tobacco smoking and alcohol abuse on drug consumption. Alcohol abuse plays a mediating role in the relationship between tobacco smoking and drug use.

## 1. Introduction

Substance abuse in young adults and university students is a frequent issue and a major public health concern. Recent research indicates that tobacco, alcohol and illegal drug consumption is highly prevalent among Spanish university students, especially regarding cannabis use [1,2,3,4,5,6]. Prevalence of tobacco smoking in this population ranges from 19% to 34% [4,5,6], and up to 27%–59% of university students in Spain demonstrate alcohol abuse and risky consumption [1,2,4,5,6,7]. During the last ten years, several studies have estimated that the prevalence of illegal drug consumption in Spanish university students is as high as 16%–42% [1,2,3,4,5,6,8], and approximately 40% of students with alcohol issues frequently consume cannabis as well [1].

Tobacco smoking, alcohol abuse, and illegal drug consumption have serious consequences on both individual and public health. Binge drinking is associated with deficits in different executive functions depending on the integrity of the dorsolateral prefrontal cortex, such as verbal and spatial working memory [9,10], difficulties in adaptation to academic life, and poorer performance at university [11,12,13,14], especially if students combine alcohol, tobacco, and cannabis consumption [1,15,16,17]. This is to be expected as cannabis use alone has major effects on several executive functions independently of alcohol abuse, like poorer inhibitory control, working memory, delayed memory and perceptual reasoning [18]. Alcohol and drug abuse in university students are risk factors for later mental health problems as well, including suicidal ideation, later substance use disorder and poorer perceived health and quality of life [13,17,19,20]. Risky sexual behavior is one of the consequences of most interest for public health, as young-adult users of alcohol and cannabis are at higher risk for engaging in unsafe sex, including sex under the influence of alcohol and sex without a condom [21,22]. Additionally, drug and alcohol use are associated with higher involvement in risky driving behavior [3].

There are several factors that contribute to explaining substance abuse in university students. Risky behaviors in higher education are heavily influenced by students’ expectations and abilities to cope with and to adjust to the highly competitive and challenging new environment [1,23,24,25,26]. Students who struggle with transitioning to university are at higher risk for substance abuse, but individual, family and environmental related factors associate with risky behaviors as well. The effects of sex roles on substance abuse is not clear, as some studies report that young men are more likely to engage in risky alcohol and drug behaviors, but others authors have reported the opposite [2,4,5,27]. Students living away from the family home (i.e., students who live with other students) are at greater risk of engaging in unhealthy lifestyles, including tobacco smoking, risky alcohol behavior and drug abuse [1,6,8,28,29]. This is of particular importance given that a high percentage of Spanish university students live away from home during the academic year [8,29,30]. While there seems to be an interaction between tobacco smoking, risky alcohol behavior and drug consumption, few studies have estimated how tobacco smoking and alcohol abuse in Spanish higher education students interrelate with environmental factors to predict drug consumption.

Apart from individual and environmental effects on substance abuse, use of tobacco and alcohol have been reported as risk factors for illegal drug consumption in higher education students in both cross-sectional and longitudinal studies [1,2,6,31,32,33,34,35,36,37,38,39,40]. Tobacco smoking increases the risk of both illicit drug use (i.e., cannabis use, illegal drug use and other non-medical use of prescription drugs) [31,32,33] and alcohol abuse [31,34,35]. Students with risky alcohol behavior are more likely to show illegal drug abuse as well [36,37]. Moreover, both tobacco smoking and alcohol abuse increase the risk of illicit drug abuse in this population [1,6,38,39,40]. Additionally, health problems occur more frequently and with more adverse consequences in polydrug using students [41]. Given that a high percentage of Spanish university students consume tobacco and show risky alcohol behavior [1,2,4,5,6,7], this situation represents a major public health issue.

Overall, previous research shows that tobacco smoking alone predicts both alcohol abuse and drug consumption. As alcohol misuse is influenced by tobacco smoking, while simultaneously increasing the risk of illicit drug use, it may be possible for alcohol abuse to play a mediating effect in the relationship between tobacco smoking and drug use in university students. Exploring this association could contribute to the design of tailored and more effective interventions aimed to prevent both legal and illegal substance consumption. However, to the best of our knowledge, this mediating effect has not been explored yet.

This study aimed to expand on previous research by exploring: (1) the prevalence of tobacco smoking, alcohol abuse, and drug consumption in Spanish university students, (2) the interrelated influence of individual and environmental factors on tobacco smoking, alcohol abuse, and drug consumption, and (3) the mediating role of tobacco smoking and alcohol abuse on drug consumption in university students.

The hypotheses of this study for the third aim are as follows:

**Hypothesis** **1** **(H1).** *Tobacco smoking significantly influences drug consumption.*

**Hypothesis** **2** **(H2).** *Tobacco smoking significantly influences alcohol abuse.*

**Hypothesis** **3** **(H3).** *Alcohol abuse significantly influences drug consumption.*

**Hypothesis** **4** **(H4).** *Alcohol abuse significantly mediates the relationship between tobacco smoking and drug consumption.*

## 2. Materials and Methods

### 2.1. Study Design, Sample and Procedures

We conducted a cross-sectional study of a random sample of students at the University of A Coruña (Spain) which holds approximately 13,600 undergraduate and 3150 graduate students. A total valid sample size of 379 was required to assess an expected prevalence of tobacco smoking of 30.1%, alcohol abuse of 26.5%, and drug consumption of 43%, assuming a significance level of α = 0.05 (95% confidence interval) and a maximum margin of sampling error of 5% [2,7,8].

An online survey was sent to all undergraduate and graduate students’ institutional e-mail addresses from February to June 2018 as part of a larger study on healthy lifestyles among University students [42]. The e-mail included information regarding the study objectives and methodology and the link to the online questionnaires. It was stated that the participation was voluntary and data was collected anonymously. This study was approved by the University Research Ethics Committee on February 2018 prior to data gathering (code 20180201).

### 2.2. Measures

Demographic and environmental data included age (in years), sex (man vs. woman), living arrangement (lives with family/partner/alone vs. lives with other students) and living area (urban, suburban or rural). Academic data included student status (bachelor student, master’s student or PhD student) and field of study (Arts and Humanities, Engineering and Architecture, Health Sciences, Sciences or Social and Legal Sciences).

Substance abuse assessment included tobacco smoking behavior, alcohol abuse and drug consumption.

#### 2.2.1. Tobacco Smoking

Students were asked to answer a question regarding their current tobacco smoking status: (1) never smoked; (2) ex-smoker (have not smoked cigarettes during at least the past twelve months); (3) current smoker (<15 cigarettes per day); and (4) current smoker (≥15 cigarettes per day). Students were then classified as non-smoker (status 1 and 2) and current smoker (status 3 and 4) for bivariate and multivariate analyses.

#### 2.2.2. Alcohol Abuse

Alcohol abuse was measured with the Spanish version of the Alcohol Use Disorders Identification Test Consumption (AUDIT-C) [7,43]. The AUDIT-C is a widely-used, three-item self-administered screening instrument for alcohol abuse that reports (1) frequency of drinking, (2) number of drinks consumed on a typical drinking day, and (3) frequency of binge-drinking. Each AUDIT-C item is scored from 0 to 4, resulting in a total score ranging from 0 to 12. According to the Spanish validation study, scores of 5 or higher in men and 4 or higher in women are considered the best cutoff points to detect alcohol abuse in Spanish university students (sensitivity: men = 91%, women = 86%; specificity: men = 84%, women = 88%) [7]. Overall, the AUDIT-C is a valid and reliable tool to identify alcohol abuse in Spanish higher education students (Cronbach’s alpha = 0.75, AUC-ROC = 0.941 for men, 0.945 for women) [7].

#### 2.2.3. Drug Consumption

Students noted their illegal drug consumption in the last six months on a list of the most-consumed illicit drugs in Spanish adults, which included cannabis, cocaine, designer drugs (MDMA-ecstasy, GHB-liquid ecstasy), amphetamines/speed, hallucinogens (LSD, ketamine, psilocybin/magic mushrooms), opioids (including heroin), legal highs, and codeine [44,45]. Participants were asked to rate whether they had consumed each drug: (0) never consumed; (1) once or twice in life but not in the last six months; (2) in the last six months; (3) in the last month; and (4) on weekends. As the study aim was to get insight about the general substance consumption of university students, participants were not asked about the frequencies of drug use.

Students were then classified in two groups on the basis of drug consumption during the last six months: non-consumers if they had not consumed any drug during the last six months, and drug consumers if they had used at least one drug during the last six months.

### 2.3. Analysis

Descriptive and inferential analyses were conducted to assess the objectives and hypotheses of the study. Prevalence rates of substance use and Odds Ratio estimates were calculated using EPIDAT 3.1. (Consellería de Sanidade, Galicia, Spain). Associations between substance use and categorical variables (i.e., sex and environmental factors) were addressed using Chi-square tests. Student *t*-test was used to explore the association between substance abuse and students’ age. Logistic regression models were conducted to identify risk factors for drug consumption. Bivariate and multivariate analyses were conducted with the SPSS version 20.0 (SPSS Inc., Chicago, IL, USA). A value of *p* < 0.05 was considered statistically significant.

#### Mediation Analysis

**Figure 1:**
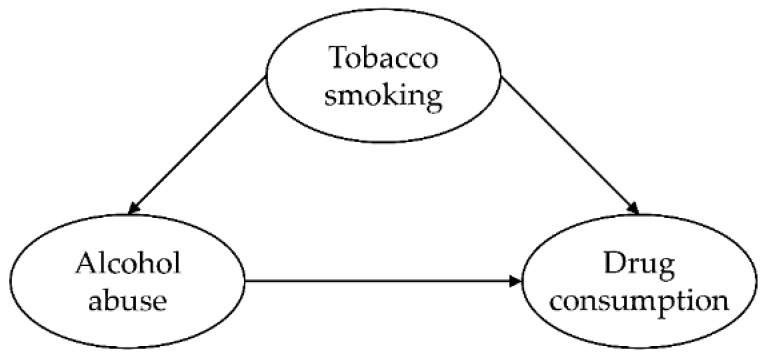
Conceptional model of the mediating effect of alcohol abuse in the relationship between tobacco smoking and drug consumption.

A Partial least squares equation modeling approach (PLS-SEM) was employed to explore the mediating role of alcohol abuse in the relationship between tobacco smoking, alcohol abuse and drug consumption. SmartPLS v. 3.2.9. (Ringle, Wende & Becker, Bönningstedt, Germany) was used to test the structural model (Figure 1). Tobacco smoking status (non-smoker vs. current smoker) and alcohol abuse (indicators: AUDIT-C items) were entered as predictor factors, and drug consumption was entered as the dependent variable (indicators: consumption of each drug during the last six months).

Path weighting scheme was run with a maximum of 300 iterations and a stop criterion of 1*10^−7^. Reliability was assessed with internal consistency (Cronbach’s alpha > 0.7) and composite reliability (>0.6). Indicators with outer loadings <0.4 were removed [46]. An average variance extracted (AVE) of >0.5 was considered as indicative of good convergent validity. The heterotrait–monotrait ratio of correlations (HTMT) was used to establish discriminant validity (HTMT < 0.9) [47]. A total of 5000 bootstrap subsamples were used to test the hypotheses. Both direct, indirect and total effects of tobacco smoking and alcohol abuse on drug consumption were estimated. Variance accounted for (VAF) values of >0.2 and >0.8 demonstrated partial and full mediation, respectively [46].

## 3. Results

Of the 17,032 students who were invited to participate in the study, 584 (3.4%) completed the questionnaire, but 34 responses were discarded (i.e., incomplete or invalid). Thus, a total sample of 550 students (94.2%) were included in the analyses (M = 23.1 years, SD = 5.4; men = 28.9%). Environmental and academic characteristics of the sample are shown in Table 1.

### 3.1. Prevalence of Substance Abuse and Associated Factors

As displayed in Table 2, 74.4% of students had never smoked, and 10.5% were ex-smokers. Up to 22.5% of the sample showed alcohol use issues. The most consumed drug was cannabis; as many as 43.6% of the students had consumed cannabis at least once or twice in their lives, and 14.9% of the students had consumed cannabis during the last six months. Prevalence of use of at least one drug during the last six months was 17.3%. The number of drugs used by those students who consumed illegal drugs during the last six months ranged from 1 to 7 (M = 1.3, SD = 0.9). Detailed information regarding AUDIT-C is shown in Table A1 (Appendix A).

Regarding risk factors for substance use (Table 3), age was associated with tobacco smoking, but not with alcohol or drug consumption. Male students reported more drug consumption than female students, and those students who lived with other students showed more alcohol and drug consumption than those students who had other living arrangements (i.e., living with family/partner or alone). Living area and academic factors were not associated with substance use.

Tobacco smoking and alcohol abuse both predicted drug consumption independently. Students who smoked tobacco cigarettes were almost six times more likely to present alcohol abuse (OR = 5.8, 95% CI = 3.6–9.5) and seven times more likely to consume drugs (OR = 7.0, 95% CI = 4.2–11.7). Additionally, those students with alcohol abuse issues were four times more likely to consume drugs (OR = 4.6, 95% CI = 2.8–7.3).

According to the multivariate analysis, predictors for drug consumption were tobacco smoking, alcohol abuse, male sex, and living with other students (Table 4).

### 3.2. Mediating Analysis

**Figure 2:**
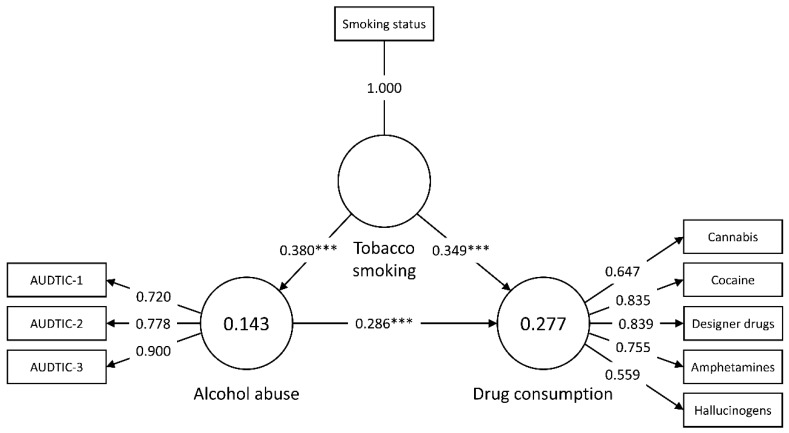
Path analysis of the mediating relationship. *** *p* < 0.001.

Codeine, legal highs, and opioids were excluded of the analysis as they did not meet the threshold limit of reliability and validity [46]. The final model of the analysis had two constructs with reflective measurements (alcohol abuse and drug consumption) and one binary construct as predictor (tobacco smoking status) (Figure 2). As Table 5 illustrates, Cronbach’s alpha, composite reliability, and AVE values met the recommended criteria. HTMT values were all below 0.9 (0.487–0.506).

The outcomes of hypotheses testing are presented in Table 6. The bootstrapping results revealed that there was a direct effect of both tobacco smoking and alcohol abuse on drug consumption during the last six months, while tobacco smoking also had a direct effect on alcohol abuse. The results of the mediating analysis supported the significant and partial effect of alcohol abuse as a mediator in the relationship between tobacco smoking and drug consumption (VAF = 24.0%).

## 4. Discussion

The main findings of this study were as follows: (1) prevalence of substance use in the sample is high, especially considering that up to 22.5% of the participants reported alcohol abuse and that almost half of the students had used cannabis at least once in their life; (2) men and students who lived with other students were more likely to consume illegal drugs; (3) tobacco smoking and risky alcohol behavior were associated with illegal drug consumption, both in the bivariate and the multivariate analyses; and (4) alcohol had a mediating effect in the relationship between tobacco smoking and illegal drug consumption in university students.

### 4.1. Prevalence of Substance Use

Rates of substance abuse in this sample are high but similar to those found in previous research on Spanish university students. Up to 15% of students were currently smokers at the time of this study, which is less than reported by other Spanish authors during the past decade [4,5,6,29]. However, these findings are in line with some recent published works on Spanish university students that indicate that tobacco consumption may be decreasing in younger university students [2,48].

As expected, alcohol was the most consumed substance, as 84.9% of students reported consuming alcohol at least once a month, and 54.2% reported regular consumption according to the AUDIT-C (i.e., at least 2–4 times a month). Alcohol use is highly prevalent among university students, both in Spain and in other European and American regions [2,16,17,28,29,38,49,50,51]. New and complex patterns of alcohol abuse exist among university students, and these problems represent a major public health concern [1,9,20,52,53].

Our findings regarding illegal drug use confirm that cannabis is the most consumed illegal drug among university students (43.6%). Almost half of students had used cannabis at least once or twice in their life, and 14.8% reported cannabis consumption during the last six months. These rates are worrying but in line with previous research on cannabis use in university students [1,2,3,4,5,6,8]. Studies regarding other illegal drugs apart from cannabis use are scarce, as most of them solely explore cannabis consumption [8,54]. However, hard drugs have pervasive and negative effects on mental health, behavior, and further drug use problems [55], and therefore should be further addressed as they seem to be prevalent in this population.

### 4.2. Individual and Environmental Risk Factors for Tobacco Smoking, Alcohol Abuse and Illegal Drug Consumption

Male and female students reported similar rates of tobacco smoking and alcohol abuse, while men were more likely to engage in illegal drug consumption. These findings are consistent with previous research that systematically reports male sex as a factor risk for the use of cannabis and other drugs [8,28,29]. Men are more likely to simultaneously co-use cannabis with alcohol and tobacco as well [56]. The well-explored association between impulsivity traits and substance consumption [55,57,58] could contribute to explain why male students use drugs more than women, as impulsivity disorders are more prevalent among men [59,60].

Findings from this study confirm that environmental factors have a great effect on substance abuse. Living with other students was a risk factor for alcohol abuse and drug consumption, even after multivariate analysis, which is in line with most studies regarding substance abuse in university students [1,6,8,28,29,51,61]. Several authors propose that family proximity plays a protective role in substance abuse among adolescents and higher education students [29,62,63,64]. This protective factor could contribute to explaining why students who live away from the family home are more likely to engage in risky alcohol behaviors and illicit drug use. However, this shielding effect could not be as effective in heavy drinking episodes or binge drinking in Spanish young adults [65], and future studies should explore the longitudinal effects of living away from the family home on risky alcohol behavior among university students.

Apart from the potential protective role that parental monitoring may play, interaction with peers contribute to explaining why living with other students is associated with substance abuse. Research has demonstrated that both peers’ substance use and peers’ positive attitude toward drugs strongly associate with tobacco smoking, alcohol abuse, and drug consumption among university students [26]. As a matter of fact, the fear of being socially alienated and excluded is one of the factors that relates the most with alcohol abuse in this population [62,66].

### 4.3. Relationship between Tobacco Smoking, Alcohol Abuse and Illegal Drug Consumption

Both tobacco smoking and alcohol abuse significantly predicted illegal drug consumption in university students, even after controlling for sex and living arrangement. Tobacco smoking was the strongest predictor for illegal drug consumption, both in the multivariate analysis and in the PLS-SEM mediating analysis. According to the mediating analysis, alcohol abuse alone had both a direct and indirect influence on drug consumption among university students. To the best of our knowledge, this analysis had not been explored before, but this finding is consistent with previous research in the relationship between tobacco smoking, alcohol abuse and drug consumption [28,38,39,67,68].

Findings from the mediating analysis could be explained by the Gateway Hypothesis, which describes how use of legal and easily accessible drugs (typically tobacco or alcohol) precedes use of harder and more addictive drugs, such as marijuana, cocaine and other illegal drugs [69]. This sequence of progression of drug consumption has been extensively explored and is well-defined, especially in Western populations [69,70]. Although this hypothesis has been discussed and is not without controversy, research supports that the gateway sequence (i.e., tobacco/alcohol use precedes the use of cannabis, which in turn precedes the use of other illegal drugs) is followed by the majority of illicit drug users [69,70,71]. Numerous retrospective and longitudinal studies have found a positive association between early exposure to cigarette smoking and alcohol use and later use of marijuana, cocaine, non-medical use of prescription drugs and other illegal drugs in humans [2,33,35,72,73,74,75].

Regarding findings from longitudinal studies, Nkansah-Amankra and Minello followed 14,738 American adolescents over 14 years and gathered data about their legal and illegal substance use at baseline (early adolescence), adolescence (12–18 years), and early (19–23 years) and young adulthood (24–33 years), considering tobacco, alcohol, and marijuana as “gateway” drugs [72,73]. Overall, they found that early use of cigarettes and alcohol increased the likelihood of using marijuana and other illegal drugs during older adolescence and early and young adulthood [72,73]. However, early use of marijuana and other illicit drugs greatly increased the risk of using harder drugs in adults older than 23 years [72]. Given that participants in our study were an average of 23 years, our findings could reflect an early exposure to “gateway” substances (i.e., tobacco and alcohol) during early or older adolescence, though this relationship cannot be established as this study is not longitudinal in nature.

The outcomes of this study may be useful for expanding on the relationship between individual and environmental factors and substance abuse, and for assessing the mediating effect of alcohol in the relationship between tobacco smoking and drug consumption. This finding is particularly relevant because alcohol abuse was the most prevalent risky behavior in the sample. Most university students consume alcohol regularly as a leisure and social activity, and new patterns of alcohol abuse are present in this population, such as binge drinking, weekend drinking and alone drinking [17,20,52,53,56]. While both tobacco and alcohol are usually proposed as the primary drivers of the gateway hypothesis or “gateway” drugs, [69,70], the mediating effect of alcohol abuse in the relationship between tobacco smoking and illegal drug consumption suggests that alcohol may play a more complex role in this hypothesis, or even represent the “gateway” drug leading to the use of other licit and/or illicit drugs [76].

Overall, risky alcohol consumption behavior has an adverse effect across psychosocial wellbeing, academic performance, quality of life, and general health [9,10,11,12,13,14,41,77,78], but also contributes to increasing the risk of drug abuse in students who already smoke cigarettes. This is of utmost importance because most adolescents and young adults believe that intermittent tobacco smoking causes little or no harm, and therefore are at more risk of engaging in risky and unhealthy behaviors [79].

Prevention programs aimed to decrease the prevalence of tobacco smoking, alcohol misuse or illegal drug consumption in university students should especially focus on students who are in a more vulnerable situation. Due to the complex interrelation between the individual and environmental factors that altogether contribute to substance abuse, a multidisciplinary approach should be used. It is necessary for health professionals and academic institutions to design collaborative prevention and intervention strategies. Given the relationship between environmental factors and substance abuse, and the impact of substance abuse on daily participation, behavior and performance, occupational therapists and psychologists could significantly contribute to the multidisciplinary team in order to promote healthy lifestyles in university students [80].

### 4.4. Limitations and Future Research Directions

This study is not without limitations. Data collection relied exclusively on self-reported questionnaires, which may introduce bias. The frequency of illegal drug use (i.e., monthly, weekly or daily) was not established, and should be comprehensively explored in future studies. Another limitation is that all participants came from one university in Northwest Spain and participation rate was low. It may be possible that those students with higher illicit drug consumption may have been less likely to respond as by doing so, they would be disclosing illegal behavior. Questionnaires were responded to anonymously and efforts were made to ensure anonymity of the participants (i.e., age was registered in years and not as birth date) to try and prevent this potential bias. Additionally, the sample included participants from different geographical settings, sociodemographics, and academic backgrounds, and findings are similar to other studies conducted with Spanish students from other regions, so it can be assumed that conclusions from this study regarding the mediating role of alcohol abuse in the relationship between tobacco smoking and illegal drug consumption can be generalized to other university students. Finally, cross-sectional studies cannot establish causality between individual and environmental factors and substance consumption.

## 5. Conclusions

Tobacco smoking, alcohol abuse, and illegal drug consumption are frequent problems in Spanish university students. Sex and living with other students interrelate with poly-substance use to predict alcohol and drug consumption. Findings from this study reveal that alcohol abuse has a mediating effect in the relationship between tobacco smoking and drug consumption in university students, and therefore risky alcohol behaviors should be promptly assessed in this population. Due to its consequences on quality of life, mental health, and academic performance, a multidisciplinary approach should be implemented in academic institutions to prevent and treat substance abuse.

## Figures and Tables

**Table 1:** Environmental and academic characteristics of the sample (*n* = 550).

Factors	*n*	%
House Arrangement		
Lives with Family	220	40.0
Lives Alone	40	7.3
Lives with Partner	57	10.4
Other	40	7.3
Lives with Other Students	193	35.1
Living Area		
Urban	302	54.9
Suburban	141	25.6
Rural	107	19.5
Student Status		
Bachelor Student	447	81.3
Master’s Student	55	10.0
PhD Student	48	8.7
Field of Study		
Arts and Humanities	28	5.1
Engineering and Architecture	142	25.8
Health Sciences	103	18.7
Sciences	76	13.8
Social and Legal Sciences	201	36.5

**Table 2:** Prevalence of substance use in students (*n* = 550).

Substance Abuse.	*n*	%
Tobacco Smoking		
Never Smoked	409	74.4
Ex-smoker	58	10.5
Current Smoker (<15 c/d)	72	13.1
Current Smoker (≥15 c/d)	11	2.0
Non-smoker	467	84.9
Current Smoker	83	15.1
Alcohol Abuse		
Low-risk Drinker	426	77.5
Alcohol Abuse	124	22.5
Drug Consumption		
Cannabis		
Never Consumed	310	56.4
Once or Twice in Life (but not in the Last Six Months)	158	28.7
In the Last Six Months	31	5.6
In the Last Month	36	6.5
On Weekends	15	2.7
Cocaine		
Never Consumed	514	93.5
Once or Twice in Life (but not in the Last Six Months)	25	4.5
In the Last Six Months	5	0.9
In the Last Month	5	0.9
On Weekends	1	0.2
Designer Drugs		
Never Consumed	512	93.1
Once or Twice in Life (but not in the Last Six Months)	29	5.3
In the Last Six Months	4	0.7
In the Last Month	4	0.7
On Weekends	1	0.2
Amphetamines/Speed		
Never Consumed	529	96.2
Once or Twice in Life (but not in the Last Six Months)	14	2.5
In the Last Six Months	4	0.7
In the Last Month	3	0.5
On Weekends	0	0.0
Hallucinogens		
Never Consumed	524	95.3
Once or Twice in Life (but not in the Last Six Months)	20	3.6
In the Last Six Months	4	0.7
In the Last Month	2	0.4
On Weekends	0	0.0
Opioids		
Never Consumed	546	99.3
Once or Twice in Life (but not in the Last Six Months)	3	0.5
In the Last Six Months	0	0.0
In the Last Month	0	0.0
On Weekends	1	0.2
Legal Highs		
Never Consumed	546	99.3
Once or Twice in Life (but not in the Last Six Months)	1	0.2
In the Last Six Months	0	0.0
In the Last Month	1	0.2
On Weekends	2	0.4
Codeine		
Never Consumed	520	94.5
Once or Twice in Life (but not in the Last Six Months)	23	4.2
In the Last Six Months	4	0.7
In the Last Month	2	0.4
On Weekends	1	0.2
Non-Consumer	455	82.7
Drug Consumer	95	17.3

**Table 3:** Sociodemographic and environmental risk factors for substance abuse (*n* = 550).

Factors	Current Smoker	*p* Value	Alcohol Abuse	*p* Value	Drug Consumption	*p* Value
Age (M (SD))	24.2 (6.7)	0.039	22.5 (4.0)	0.230	22.9 (6.0)	0.726
Sex (N (%))		0.792		0.067		0.002
Men	25 (15.7)		44 (27.7)		40 (25.2)	
Women	58 (14.8)		80 (20.5)		55 (14.1)	
Living Arrangement (N (%))		0.473		0.025		0.003
Lives with Other Students	32 (16.6)		54 (28.0)		46 (23.8)	
Lives with Family/Partner/Alone	51 (14.3)		70 (19.6)		49 (13.7)	
Living Area (N (%))		0.115		0.675		0.252
Urban	49 (16.2)		71 (23.5)		56 (18.5)	
Suburban	14 (9.9)		28 (19.9)		18 (12.8)	
Rural	20 (18.7)		25 (23.4)		21 (19.6)	
Student Status (N (%))		0.540		0.213		0.082
Bachelor Student	68 (15.2)		101 (22.6)		80 (17.9)	
Master’s Student	10 (18.2)		16 (29.1)		12 (21.8)	
PhD Student	5 (10.4)		7 (14.6)		3 (6.2)	
Field of Study (N (%))		0.891		0.277		0.366
Arts and Humanities	4 (14.3)		3 (10.7)		4 (14.3)	
Engineering and Architecture	18 (12.7)		29 (20.4)		38 (26.8)	
Health Sciences	17 (16.5)		16 (15.5)		21 (20.4)	
Sciences	11 (14.5)		8 (10.5)		13 (17.1)	
Social and Legal Sciences	33 (16.4)		39 (19.4)		48 (23.9)	
Substance Abuse (N (%))						
Current Smoker	-	-	45 (54.2)	<0.001	40 (48.2)	<0.001
Non-Smoker	-	-	79 (16.9)		55 (11.8)	
Alcohol Abuse	45 (36.3)	<0.001	-	-	46 (37.1)	<0.001
Low-Risk Drinker	38 (8.9)		-	-	49 (11.5)	
Drug Consumption	40 (42.1)	<0.001	46 (48.4)	<0.001	-	-
Non-Consumer	43 (9.5)		78 (17.1)		-	-

**Table 4:** Logistic multivariate analysis to identify multifactorial predictors for drug consumption.

Variable	B	SE	*p* Value	OR (95% CI)
Tobacco Smoking	1.667	0.289	<0.001	5.3 (3.0–9.3)
Alcohol Abuse	0.989	0.269	<0.001	2.7 (1.6–4.6)
Male Sex	0.713	0.259	0.006	2.0 (1.2–3.4)
Lives with Other Students	0.601	0.253	0.017	1.8 (1.1–3.0)

B = B coefficient value; SE = standard error; OR = odds ratio; 95% CI = 95% confidence interval.

**Table 5:** Assessment of the measurement model (reflective).

Construct	Items	LV	α	CR	AVE
Alcohol Abuse			0.756	0.844	0.645
	AUDITC-1	0.720			
	AUDITC-2	0.778			
	AUDITC-3	0.900			
Drug Consumption			0.786	0.852	0.541
	Cannabis	0.647			
	Cocaine	0.835			
	Designer Drugs	0.839			
	Amphetamines	0.755			
	Hallucinogens	0.559			

LV = outer loading value; α = Cronbach’s alpha; CR = composite reliability; AVE = average variance extracted.

**Table 6:** Hypotheses testing for direct and mediating relationships.

Hypotheses	Effects		*t*-Value	*p* Value	Supported
(H1) TS → DC	Path coeff.	0.349	6.335	<0.001	Yes
(H_2_) TS → AA	Path coeff.	0.380	8.146	<0.001	Yes
(H_3_) AA → DC	Path coeff.	0.286	5.035	<0.001	Yes
(H_4_) TS → AA → DC	Direct	0.349	6.335	<0.001	Yes
	Indirect	0.109	4.168	<0.001	
	Total	0.458	10.491	<0.001	

TS = tobacco smoking; AA = alcohol abuse; DU = drug consumption.

## Appendix A

**Table A1:** AUDIT-C (*n* = 550).

Audlt-C	*n*	%
How Often Do You Have a Drink Containing Alcohol?		
Never	83	15.1
Monthly or Less	169	30.7
2–4 times a month	232	42.2
2–3 times a week	55	10.0
4 or more times a week	11	2.0
How Many Standard Drinks Containing Alcohol Do You Have on a Typical Day?		
1 or 2	383	69.6
3 or 4	116	21.1
5 or 6	43	7.8
7 or 9	5	0.9
10 or more	3	0.5
How Often Do You Have Six or More Drinks on one Occasion?		
Never	335	60.9
Less than Monthly	131	23.8
Monthly	55	10.0
Weekly	28	5.1
Daily or Almost Daily	1	0.2
